# Macrophage Receptor with Collagenous Structure (MARCO) Is Processed by either Macropinocytosis or Endocytosis-Autophagy Pathway

**DOI:** 10.1371/journal.pone.0142062

**Published:** 2015-11-06

**Authors:** Seishiro Hirano, Sanae Kanno

**Affiliations:** 1 NanoTox, RCER, National Institute for Environmental Studies, 16–2 Onogawa, Tsukuba, Ibaraki 305–8506, Japan; 2 Legal Medicine, St. Marianna University School of Medicine, 2-16-1 Sugao, Miyamae-ku, Kawasaki, Kanagawa 216–8511, Japan; IISER-TVM, INDIA

## Abstract

The Macrophage Receptor with COllagenous structure (MARCO) protein is a plasma membrane receptor for un-opsonized or environmental particles on phagocytic cells. Here, we show that MARCO was internalized either by ruffling of plasma membrane followed by macropinocytosis or by endocytosis followed by fusion with autophagosome in CHO-K1 cells stably transfected with GFP-MARCO. The macropinocytic process generated large vesicles when the plasma membrane subsided. The endocytosis/autophagosome (amphisome) generated small fluorescent puncta which were visible in the presence of glutamine, chloroquine, bafilomycin, ammonia, and other amines. The small puncta, but not the large vesicles, co-localized with LC3B and lysosomes. The LC3-II/LC3-I ratio increased in the presence of glutamine, ammonia, and chloroquine in various cells. The small puncta trafficked between the peri-nuclear region and the distal ends of cells back and forth at rates of up to 2–3 μm/sec; tubulin, but not actin, regulated the trafficking of the small puncta. Besides phagocytosis MARCO, an adhesive plasma membrane receptor, may play a role in incorporation of various extracellular materials into the cell via both macropinocytic and endocytic pathways.

## Introduction

Particles opsonized with IgG or with a complement protein (C3bi) are phagocytosed by macrophages via FcγR or CR3, respectively. In addition to the receptor-mediated internalization of opsonized particles and microorganisms, macrophages recognize and take up non-opsonized or environmental particles such as silica, iron oxide, carbon soot, and polystyrene beads via Macrophage Receptor with COllagenous structure (MARCO) protein [1, 2]. The cytosolic domain of MARCO is very short [3] and no signal transduction pathway via MARCO has been proposed yet. Moreover, the metabolic fate of this plasma membrane protein has not been reported, although MARCO is known to play a pivotal role in the uptake of non-opsonized particles [4].

Canonical macroautophagy is a catabolic process in which cytosolic components, including organelles, are transported and processed in double membrane vesicles [5] and autophagy regulates cell death both positively and negatively [6]. Autophagy is also an immunologically regulated process and induction of autophagy colocalized mycobacterial phagosomes with Light Chain 3 (LC3) and consequently suppressed intracellular survival of mycobacteria in macrophages [7]. The versatile functions of autophagic molecules [8, 9] and the origin of autophagic vesicles [10–12] are still enigmatic; endoplasmic reticulum, Golgi apparatus, mitochondria, and plasma membrane have been proposed as possible sources of early autophagic vesicles [13–15].

LC3-associated phagocytosis (LAP) is a noncanonical autophagy process where components of autophagy pathway are co-opted for lysosomal degradation of phagocytosed cargos [16]. Toll-like receptor (TLR) -mediated phagocytosis of zymosan and subsequent signaling processes recruit LC3 into the single membrane of phagosomes [17]. Knockdown of ATG5 remarkably reduced LC3 recruitment to the zymosan-containing phagosomes, and LC3 was not associated with the phagosomes in ATG7-deficient mouse macrophages. Those results indicated that classic autophagy molecules are involved in TLR-mediated phagocytosis. In addition class A scavenger receptors, macrophage scavenger receptor 1 (MSR1) and MARCO, were upregulated in autophagy-deficient (*Atg7* ^-/-^) mice, suggesting that autophagy regulates phagocytosis [18].

Lipopolysaccharide (LPS) induces autophagy via Toll-like receptor 4 in RAW264.7 and human alveolar macrophages. After exposure to *Mycobacterium tuberculosis*, the bacilli were found in single-membrane phagosomes in untreated macrophages. In contrast they were found in double-membrane autophagosomes in LPS-induced autophagic macrophages [19]. Recently, it has been shown that autophagic proteins such as LC3, ATG5, and ATG7 target single membranes as well as autophagosomal double membranes [20]. The targeted single membranes originated from epithelial endocytosis, phagocytosis of bacteria, and macropinocytosis. However, phagocytosis of uncoated latex beads does not trigger LC3 recruitment to the membranes. The recruitment of autophagic proteins to single membranes is independent of mTOR activation. It has also been reported that clathrin-mediated endocytosis provides plasma membrane for the formation of pre-autophagic structures [14]. It is therefore likely that phagocytic receptors are to be metabolically processed by non-phagocytic pathways including autophagy.

In the present study, we report that MARCO is internalized by either macropinocytic or endocytic pathways. We further demonstrate that MARCO processed via the endocytic pathway subsequently is merged with the autophagic cascade, producing autophagic puncta that are regulated by glutamine or its metabolic product, ammonia.

## Materials and Methods

### Chemicals

Chemical compounds and kits used in the present study were obtained as follows: ammonium carbonate, saponin, and buffer solutions from Wako Chemical (Osaka, Japan); dinasore, bafilomycin A1, rapamycin, and trimethylamine solution from Sigma Aldrich (St. Louis, MO); trimethylamine hydrochloride from TCI (Tokyo, Japan); pHrodo^TM^Red AM intracellular pH indicator and chloroquine from Life Technologies (Carlsbad, CA); BCA protein assay kit, cytochalasin D, and nocodazole from Thermo (Waltham, MA); WST-8 Cell Counting kit from Dojindo (Osaka, Japan).

### Cells

Preparation of the plasmid construct was reported elsewhere [21]. Briefly, the murine MARCO (mMARCO)–encoding gene was engineered first into pCR8/GW/TOPO and then into pcDNA6.2/N-EmGFP using the Gateway^®^ LR Clonase Enzyme Mix (Life Technologies) according to the manufacturer’s instructions. Chinese hamster ovary (CHO) -K1 cells and human embryonic kidney HEK293 cells were stably transfected with GFP-MARCO using Lipofectamine LTX-PLUS (Life Technologies). Unless otherwise mentioned, GFP-MARCO-CHO cells were cultured in F12 medium and HEK293 and GFP-MARCO-HEK cells were cultured in DMEM medium containing 100 units/mL penicillin, 100 μg/mL streptomycin, 20 μg/mL blasticidin-S, and 10% heat-inactivated fetal bovine serum (FBS). Jurkat and J774.1 cells were cultured in RPMI1640 medium containing 100 units/mL penicillin, 100 μg/mL streptomycin, and 10% FBS.

#### Transfection of the cells with LC3B-RFP

GFP-MARCO-CHO cells were transiently transfected with LC3B-RFP or its inactive mutant LC3B(G120A)-RFP using BacMam (Baculovirus with a Mammalian promoter) reagents (Premo^TM^ Autophagy Sensor, Life Technologies). The live cells were observed by laser confocal microscopy (TCS-SP5, Leica Microsystems, Solms, Germany) or fluorescence microscopy (Eclipse TS100, Nikon, Tokyo, Japan) at 16 hr after treatment with 50 μM choloroquine.

### Western blot

GFP-MARCO-CHO, GFP-MARCO-HEK, Jurkat, and J774.1 cells were washed with phosphate-buffered saline (PBS) and lysed with RIPA buffer containing protease and phosphatase inhibitor cocktails (Halt^®^, Thermo Fisher). For western blotting the protein concentration of the lysate was adjusted at 1 mg/ml and the proteins were resolved by SDS-PAGE and then electroblotted onto a PVDF membrane. The following antibodies were used for western blot analysis using ECL (GE Healthcare, Buckinghamshire, UK): polyclonal goat-anti-GFP, and POD-conjugated anti-murine and anti-goat secondary antibodies (Santa Cruz Biotechnology, Dallas, TX); anti-LC3 antibody (8E10, human 1-120aa), anti-ATG5 antibody (4D1, human full length ATG5), and HRP-tagged anti-α-tubulin antibody (MBL, Nagoya, Japan).

### Fluorescent staining

GFP-MARCO-CHO cells cultured in an 8-well glass chamber slide were fixed in phosphate-buffered 3.7% formaldehyde solution and then permeabilized with 0.1% Triton X-100. The slides were pre-incubated in 1% bovine serum albumin (BSA), washed in PBS, and stained with rhodamine phalloidin (Life Technologies) or anti-α-tubulin antibody followed by Alexa Fluor® 594-labeled goat anti-mouse IgG with enhancer solution (Life Technologies). Samples were mounted onto slides using a DAPI-containing anti-fade reagent (ProLong Gold®; Life Technologies) and the cells were observed by fluorescence microscopy. For immunostaining with anti-hamster LAMP2 antibody, the cells were permeabilized with PBS containing 0.1% saponin and 3% BSA, with subsequent processing as described above. The anti-LAMP-2 antibody was purified from the culture medium of the hybridoma (UH3, DSHB, Iowa City, IA) using the MAbTrap^TM^ Kit (GE Healthcare). The cells were observed by fluorescence microscopy (Eclipse 80i, Nikon, Tokyo, Japan).

### Cell growth

GFP-MARCO-CHO cells were suspended in complete F12 medium at 2 x 10^4^ cells/mL and aliquotted at 100 μL/well in a 96-well culture dish. The cells were pre-cultured for 15 hr and then cultured in various conditions for 24 or 48 hr. The cell monolayers were washed twice with HBSS and the number of viable cells was evaluated by a modified MTT assay method using the WST-8 Cell Counting kit. After chromophore development, the reaction was quenched by adding one-tenth volume of 0.1 mol/L HCl solution. The O.D. at 450 nm was measured using a microplate reader (POLARstar OPTIMA, BMG Labtech, Offenburg, Germany).

### Transmission electron microscopy (TEM)

GFP-MARCO-CHO cells were cultured on a cover slip placed in a culture dish and incubated under various conditions. The cells were pre-fixed for 2 hr in 2.5% glutaraldehyde in 0.1 M phosphate buffer containing 0.2 M sucrose and post-fixed with 1% osmium tetroxide for 1 hr. After sequential dehydration using increasing concentrations of ethanol, samples were incubated in butyl glycidyl ether (BGE) and then embedded in Quetol 812 resin in a BEEM^®^ capsule (Okenshoji, Tokyo, Japan). Thin sections (80–100 nm) were prepared and stained with uranium acetate and lead citrate. The samples were observed using a transmission electron microscope (JEM-2010, JEOL, Tokyo, Japan).

### Scanning electron microscopy (SEM)

GFP-MARCO-CHO cells were pre-fixed in 2.5% glutaraldehyde and post-fixed with 1% osmium tetroxide as described above. After sequential dehydration using increasing concentrations of ethanol, the samples were subjected to critical point drying and sputter-coated with Pt/Pd. The cells were observed by scanning electron microscope (JSM-5800, JEOL).

### Statistical analyses

Cell growth data, LC3-II/LC-I ratios, and densitometric data were presented as mean ± SEM. Statistical analyses were performed by Analysis of Variance (ANOVA) followed by Tukey’s post-hoc comparison. Statistical significance was accepted when p <0.05.

## Results

### Macropinocytic vesicles and glutamine-induced small puncta

**Fig 1:**
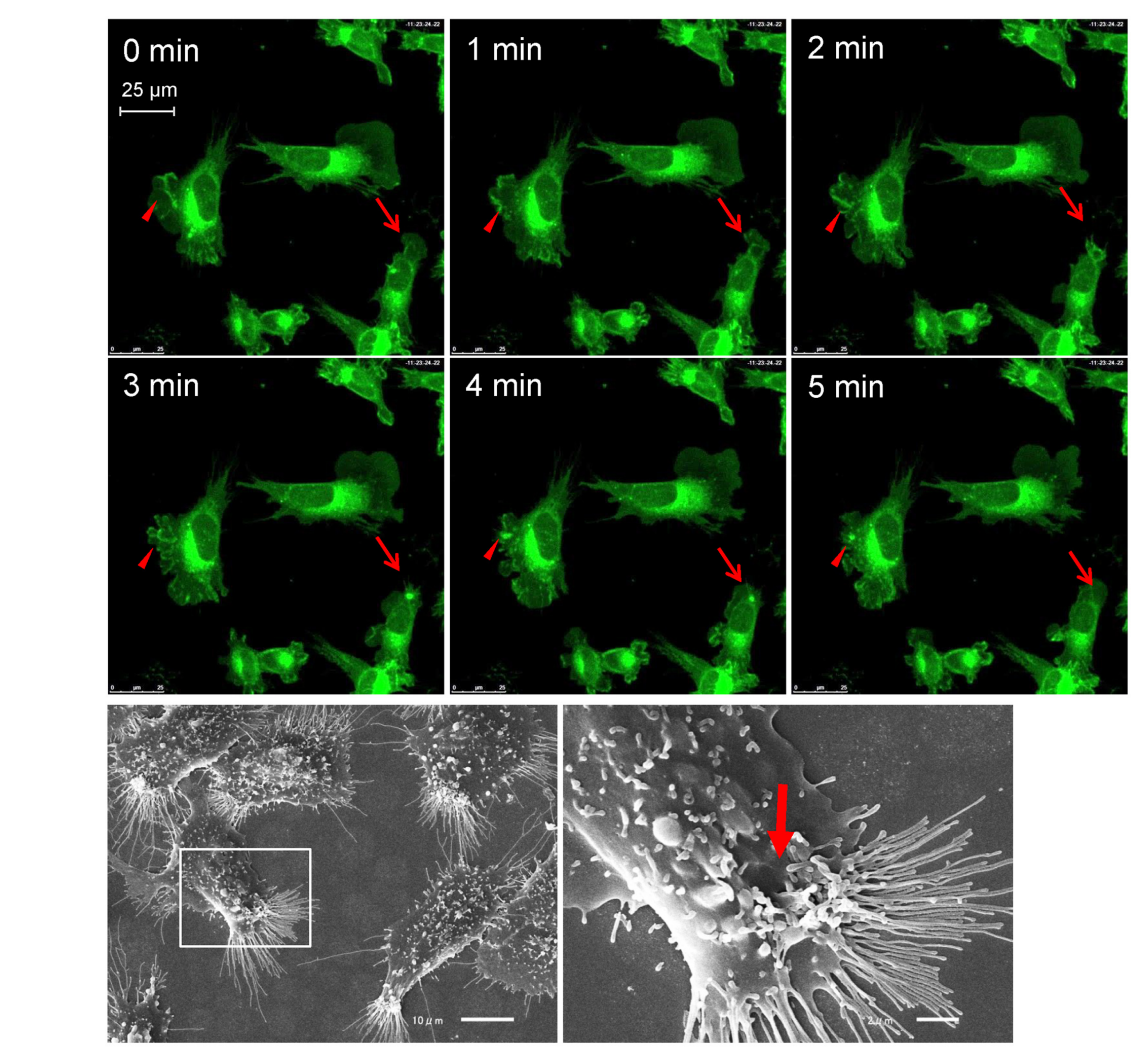
Macropinocytic activity of MARCO-GFP CHO cells. (A) The cells were grown in glass-bottom culture dishes in F12 complete medium and observed using confocal microscopy. Fluorescence images were recorded at 1-min intervals. The arrows and arrowheads indicate the formation and fate of two separate macropinocytic sites. See also S1 Movie. (B) Scanning electron micrographs of GFP-MARCO-CHO cells. The right panel provides a higher magnification image of the boxed area indicated in the left panel. The arrow indicates the macropinocytic structure.

GFP-MARCO was internalized by macropinocytosis in GFP-MARCO-CHO cells, as shown in Fig 1A and 1B and S1 Movie. Dense GFP labeling or local ruffling of plasma membrane appeared in peripheral sites of the cell as plasma membrane ruffled and the GFP-labeled membrane was pinched off and internalized as vesicles. The vesicles disappeared within several minutes.

**Fig 2:**
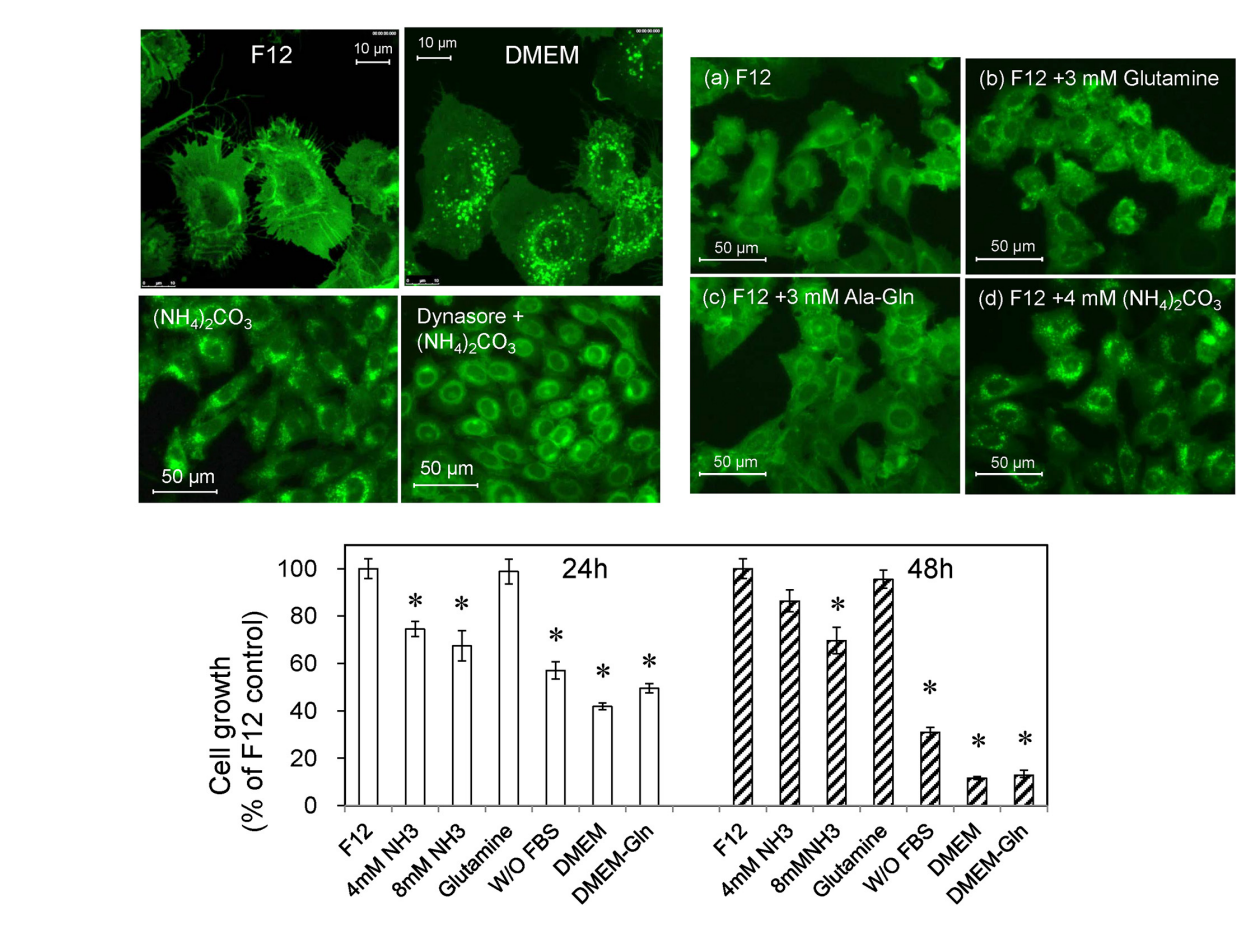
L-Glutamine and ammonia induce fluorescent puncta in GFP-MARCO-CHO cells. (A) The cells were cultured in either F12 complete medium or DMEM complete medium and the fluorescence images were captured by confocal microscopy. Many puncta (200–600 nm in diameter) appeared when the cells were culture in DMEM. (B) The cells were cultured for 12 hr in (a) F12 complete medium (containing 1 mM L-glutamine), (b) F12 medium supplemented with 3 mM L-glutamine (to a final concentration of 4 mM L-glutamine), (c) F12 medium supplemented with 3 mM L-alanyl-L-glutamine (Ala-Gln), or (d) F12 medium supplemented with 4 mM (NH_4_)_2_CO_3_. (C) Proliferation of GFP-MARCO-CHO cells in different culture media. The cells were suspended in F12 medium at 2.0 x 10^4^ cells /mL, aliquotted at 100 μL/well in a 96-well culture dish, and pre-cultured overnight. The medium then was replaced with fresh medium as follows: F12 medium; F12 medium supplemented with 4 or 8 mM (NH_4_)_2_CO_3_; F12 containing 4 mM L-glutamine, F12 without 10% FBS; DMEM; or L-glutamine-free DMEM. Cells then were cultured for 24 h or 48 h. The viable cells were assayed using WST-8. Data are presented as mean ± SEM of 6 wells per medium per time point. *, Significantly different from control (F12) value. (D) Loss of ammonia-induced fluorescent small puncta upon inhibition of the endocytic pathway. The cells were cultured in F12 culture medium and exposed to 4 mM ammonium carbonate for 6 hr in the absence or presence of 100 μM dynasore, a dynamin inhibitor.

The cells were cultured either in F12 or DMEM culture medium overnight and the live cells were observed by confocal microscopy. Surprisingly, small fluorescent puncta appeared when the cells were cultured in DMEM (Fig 2A). We examined all nutrients and supplements contained differentially between F12 and DMEM and found that the higher concentration of glutamine (4 mM) in DMEM was responsible for the formation of the small fluorescent puncta. The small fluorescent puncta (ca. 0.5 μm) observed in DMEM-grown GFP-CHO-MARCO cells were distinct from the large macropinocytic retractive vesicles (ca. 3 μm) (Fig 1A). Glutamine is catabolized to glutamate by glutaminase, and glutamate is catabolized to α–ketoglutarate by glutamate dehydrogenase, generating two ammonia molecules per glutamine in a process termed glutaminolysis. The small fluorescent puncta appeared when the cells were cultured in F12 culture medium supplemented with additional glutamine (final concentration 4 mM) or with 4 mM (NH_3_)_2_CO_3_, although supplementation of F12 with 3 mM L-alanyl-L-glutamine did not induce the puncta (Fig 2B). We additionally noted that the proliferation of GFP-MARCO-CHO cells was significantly reduced when cultured in DMEM (Fig 2C). However, 4 mM glutamine did not change the growth rate significantly, suggesting that the generation of small fluorescent puncta was independent of the cell proliferation. Endocytosis was involved in generation of the fluorescent small puncta because dynasore, an inhibitor of dynamin, reduced the number of ammonia-induced small puncta (Fig 2D).

### Glutamine-induced small puncta are related to autophagosome generated by lysosomal dysfunction

**Fig 3:**
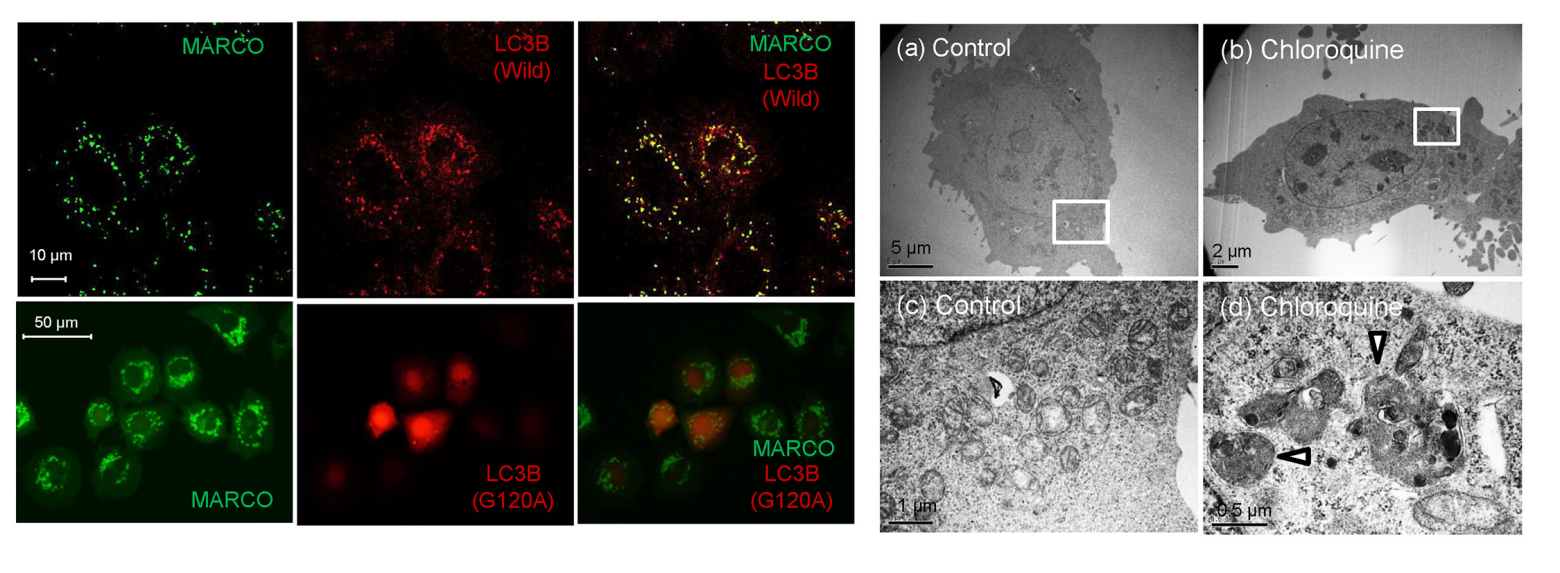
Effects of chloroquine on fluorescent puncta formation in GFP-MARCO-CHO cells. (A) Co-localization of green fluorescent puncta with LC3B. GFP-MARCO-CHO cells were transfected with constructs encoding LC3B (wild-type or G120A mutant), pre-cultured overnight in F12 complete medium, and further cultured for 16 hr in F12 complete medium supplemented with 50 μM chloroquine. (B) Transmission electron micrographs of GFP- MARCO-CHO cells. The cells were cultured for 6 hr with (b, d) or without (a, c) 50 μM chloroquine. Fig 3B(c) and (d) provide higher magnification images of the boxed areas in (a) and (b), respectively. The arrowheads indicate autophagosomes.

**Fig 4:**
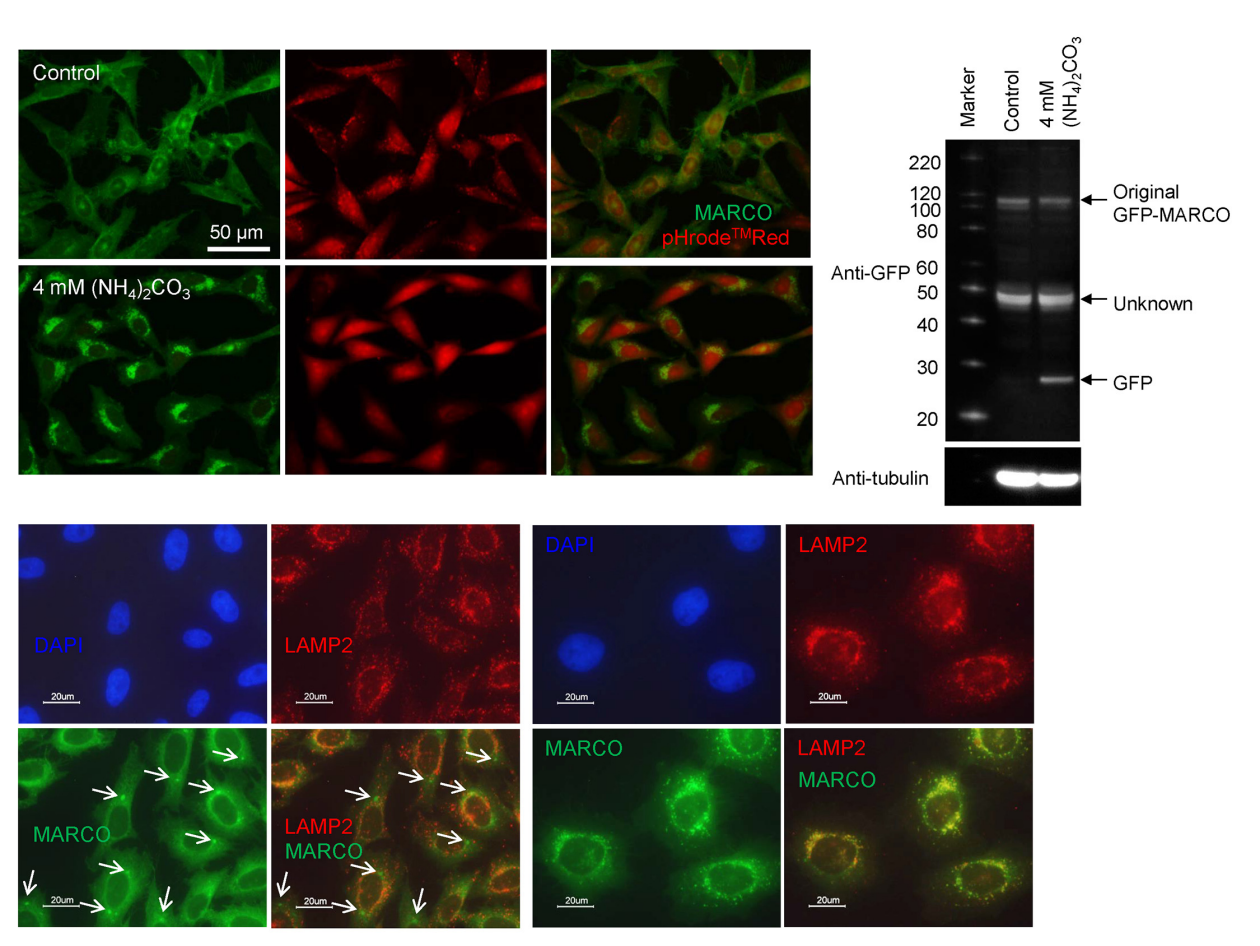
Processing of GFP-MARCO in lysosomes. (A) GFP-MARCO-CHO cells were incubated with or without (NH_4_)_2_CO_3_ and the cells were loaded with pHrode Red AM. The cells were washed and cultured in HEPES-buffered HBSS for 2h. The acidity of lysosomes was neutralized by (NH_4_)_2_CO_3_. (B) Macropinocytic inclusions (arrows) did not co-localize with lysosomes. The control GFP-MARCO-CHO cells were fixed and stained with anti-LAMP2 antibody (2^nd^ Ab: Alexa Fluor® 594-labeled goat anti-mouse IgG). (C) Co-localization of GFP-MARCO and LAMP2 in autophagic puncta. The cells were grown for 15 hr in the presence of 50 μM chloroquine. See also the legend to Fig 4 (B) for immunofluorescent staining. (D) GFP-MARCO-CHO cells were cultured for 15 hr in fresh F12 medium in the presence or absence (Control) of 4 mM (NH_4_)_2_CO_3_. The supernatant of the cellular lysate was analyzed by SDS-PAGE followed by western blotting for the detection of GFP. The membrane was reprobed with POD-tagged anti-tubulin to serve loading control.

The small fluorescent puncta induced by glutamine or ammnonia were autophagic puncta, because chloroquine, an autophagy inhibitor, also induced those small fluorescent puncta in GFP-MARCO-CHO cells cultured in F12 medium and the puncta were co-localized with wild-type LC3B, an autophagosome marker (Fig 3A). Those small puncta (ca. 0.5 μm) had a double-membrane structure as observed by TEM (Fig 3B). Bafilomycin, a specific inhibitor of vacuolar-type H^+^-ATPase and another autophagy inhibitor, also induced the fluorescent small puncta (S1 Fig). Ammonia is membrane-permeable and neutralize the acidity of lysosomes. The lysosomes were distinctly stained by pHrode^TM^Red. However, they are not differentially stained in the presence of (NH_4_)_2_CO_3_, indicating that the GFP-derived fluorescent small puncta were produced by lysosomal alkalinization (Fig 4A). The fluorescent small puncta also co-localized with lysosomal protein LAMP-2 (Fig 4C), while macropinocytic vesicles were not co-localized with LAMP-2-containing vesicles (Fig 4B), suggesting that GFP-MARCO internalized by macropinocytosis is probably recycled without digestion in lysosomes and GFP-MARCO internalized by endocytosis eventually merged with the lysosome regardless of the function of this organelle.

It is known that GFP is resistant to lysosomal proteolysis and appearance of the free GFP moiety reflects cargo delivery of GFP-LC3 from autophagosome to lysosome [22]. The appearance of a band at 27 kD in the (NH_3_)_2_CO_3_-exposed cells, which is corresponding to the free GFP moiety, indicates that the lysosomal function was compromised by alkalinization and GFP cleaved off GFP-MARCO was not digested in lysosomes (Fig 4D).

### Trafficking of ammonia-induced autophagic puncta

**Fig 5:**
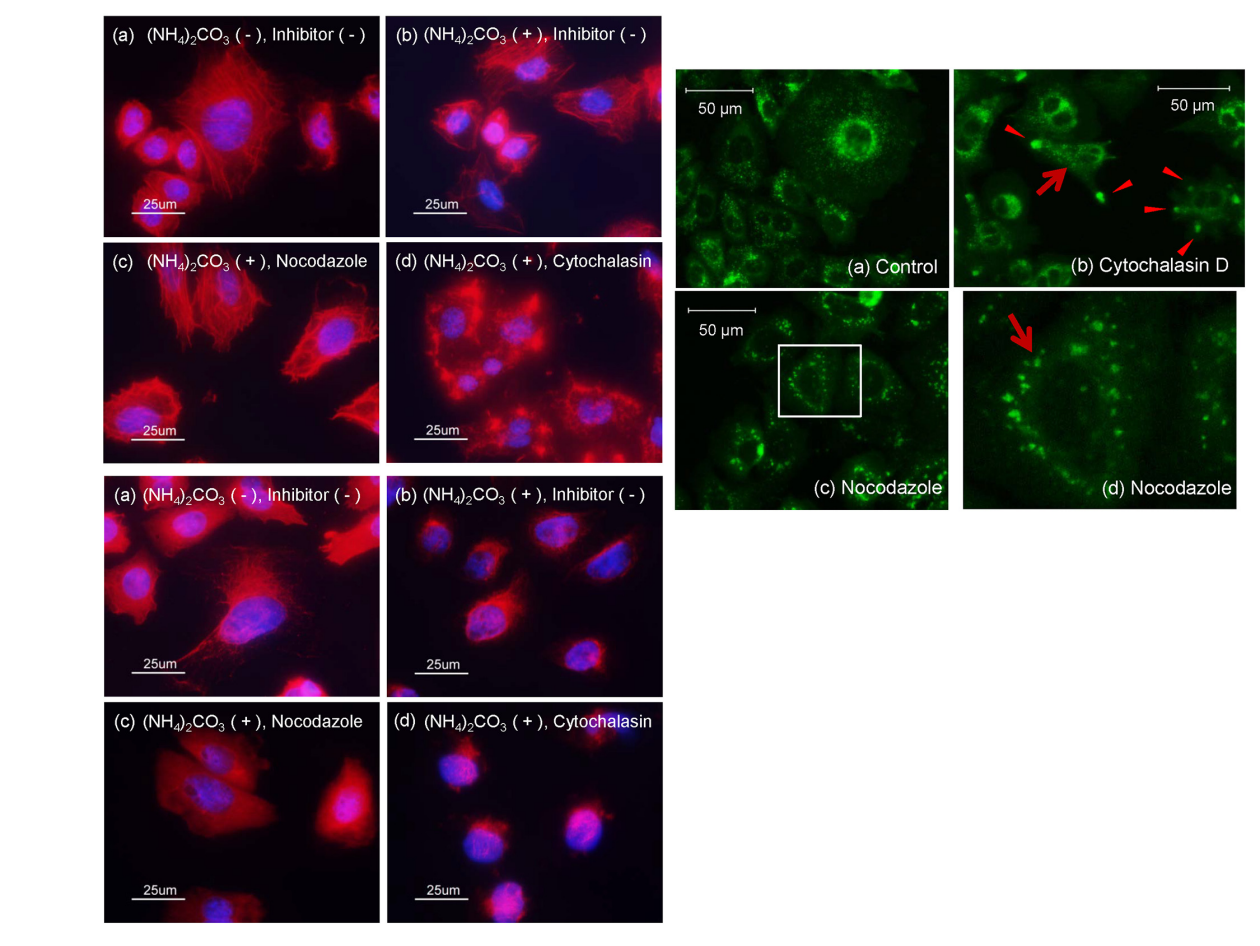
Effects of cytochalasin D and nocodazole on fluorescent autophagic puncta in GFP-MARCO-CHO cells. (A) Fluorescence staining of actin in GFP-MARCO-CHO cells. The cells were cultured for 15 hr in the absence (a) or presence of 4 mM (NH_4_)_3_CO_3_ (b-d), as follows: (b) without further stimulation; (c) with 1 μM nocodazole; or (d) with 1 μg/mL cytochalasin D. They were fixed with formalin solution and stained with rhodamine phalloidin and DAPI using standard immunofluorescence techniques. (B) Fluorescence staining of tubulin in GFP-MARCO-CHO cells. The cells were cultured and treated with chemicals as shown in Fig 5(A). They were fixed with formalin solution and treated with anti-α-tubulin followed by Alexa Fluor^®^ 594-conjugated secondary antibody and DAPI using standard immunofluorescence techniques. (C) The cells were cultured in the presence of 4 mM (NH_4_)_3_CO_3_ for 15 hr as follows; (a) without further stimulation, (b) with 1 μg/mL cytochalasin D, or (c) with 1 μM nocodazole. Fig 5C(d) provides a higher magnification image of the boxed area in (c). The arrows and arrowheads indicate autophagic puncta and interrupted macropinocytosis, respectively.

Neither inhibition of actin polymerization by cytochalasin D nor inhibition of microtubule polymerization by nocodazole was affected by (NH_4_)_2_CO_3_ (Fig 5A and 5B). Cytochalasin D crippled withdrawal movement of macropinocytosis, leaving several retreating macropinocytic vesicles at peripheral sites (Fig 5C). However, small autophagic puncta were not affected by treatment with cytochalasin D. In contrast, most autophagic small puncta agglomerated in the peri-nuclear region in cells treated with nocodazole, an inhibitor of microtubule polymerization (Fig 5C). Some autophagic small puncta moved between the peripheral and the peri-nuclear regions at speeds of up to 2–3 μm/sec. However, most of the puncta moved more slowly (S2 Movie). It is plausible that the movement of autophagic small puncta was hindered by nocodazole and the puncta were accumulated around the nucleus. Those results also suggest that macropinocytosis, which requires polymerization of actin, is not involved in the formation of autophagic puncta, if any.

### Conversion of LC3-I to LC3-II in ammonia- and amine-exposed cells

**Fig 6:**
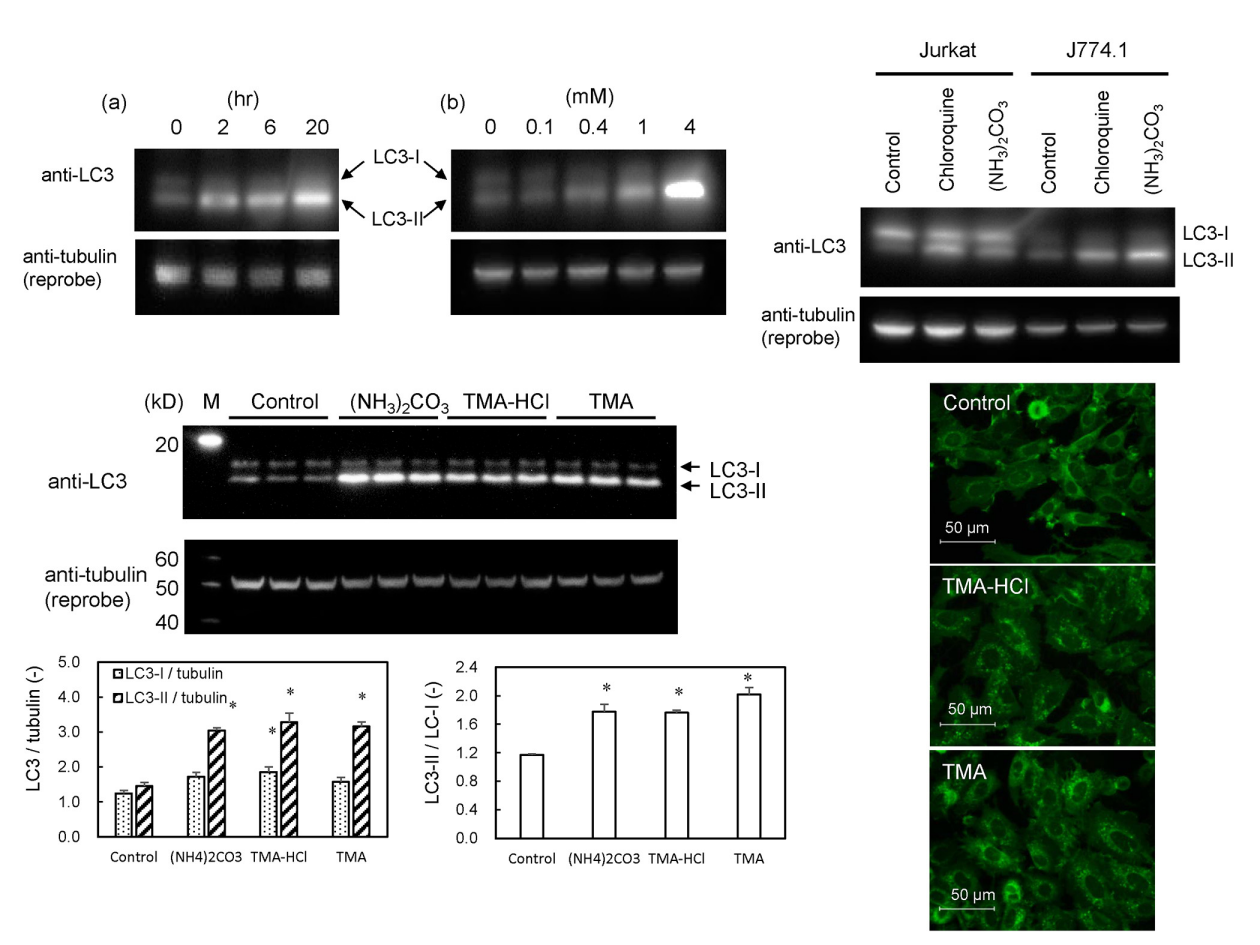
Effects of ammonia and amine compounds on conversion of LC3-I to LC3-II (lipidated LC3-I) and autophagic puncta formation. (A) Western blot analysis for the detection of LC3-I and LC3-II. GFP-MARCO-CHO cells were cultured in F12 culture medium and exposed to (NH_4_)_3_CO_3_. (a) The cells were cultured in the presence of 4 mM (NH_4_)_2_CO_3_ for 0, 2, 6, and 20 hr. (b) The cells were cultured for 6 hr in the presence of 0, 0.1, 0.4, 1, and 4 mM (NH_4_)_2_CO_3_. α-Tubulin was adopted as loading control. The band for LC3-I was not clear in GFP-MARCO-CHO cells. (B) Jurkat (human T cell leukemia) and J774.1 (murine macrophages) cells were exposed for 6 hr to 4 mM (NH_4_)_2_CO_3_ or 50 μM chloroquine. (C) GFP-MARCO-CHO cells were grown for 8 hr in the absence (Control) or presence of 4 mM (NH_4_)_2_CO_3_, 8 mM trimethylamine hydrochloride (TMA-HCl), or 8 mM trimethylamine (TMA). M, Western marker lane. α-Tubulin was adopted as loading control and the amount of LC3-I, LC3-II and tubulin were measured by densitometry. LC3-I/tubulin, LC3-II/tubulin, and LC3-II/LC3-I ratios were presented as mean ± SEM (N = 3). *, Significantly diferent from the control value. (D) Formation of fluorescent autophagic puncta by amines. GFP-MARCO-CHO cells were cultured for 8 hr in complete F12 culture medium in the absence (Control) or presence of 8 mM trimethylamine hydrochloride (TMA-HCl) or of 8 mM trimethylamine (TMA).

The conversion of LC3-I to its lipidated form LC3-II is a benchmark for autophagosomal maturation. Fig 6A shows that the lipidation of LC3 occurred in GFP-MARCO-CHO cells grown in F12 supplemented with (NH_4_)_3_CO_3_ time- and dose-dependently. The LC3 lipidation in (NH_4_)_3_CO_3_- or chloroquine-supplemented medium was observed in other types of cells such as Jurkat and J774.1 cells (Fig 6B). It should be noted that the amount of LC3 varies among the cell lines and LC3-I was not remarkably changed as LC3-II was increased clearly by (NH_4_)_3_CO_3_ and chloroquine. LC3 was lipidated in GFP-MARCO-CHO cells grown in medium supplemented with other amines such as trimethylamine (8 mM) and trimethylamine hydrochloride (8 mM) (Fig 6C). Those amines also induced autophagic small puncta (Fig 6D). Rapamycin, an inhibitor of mTOR, neither induced autophagic small puncta nor attenuated a potency of (NH_4_)_3_CO_3_ to induce formation of the puncta (S2 Fig).

**Fig 7:**
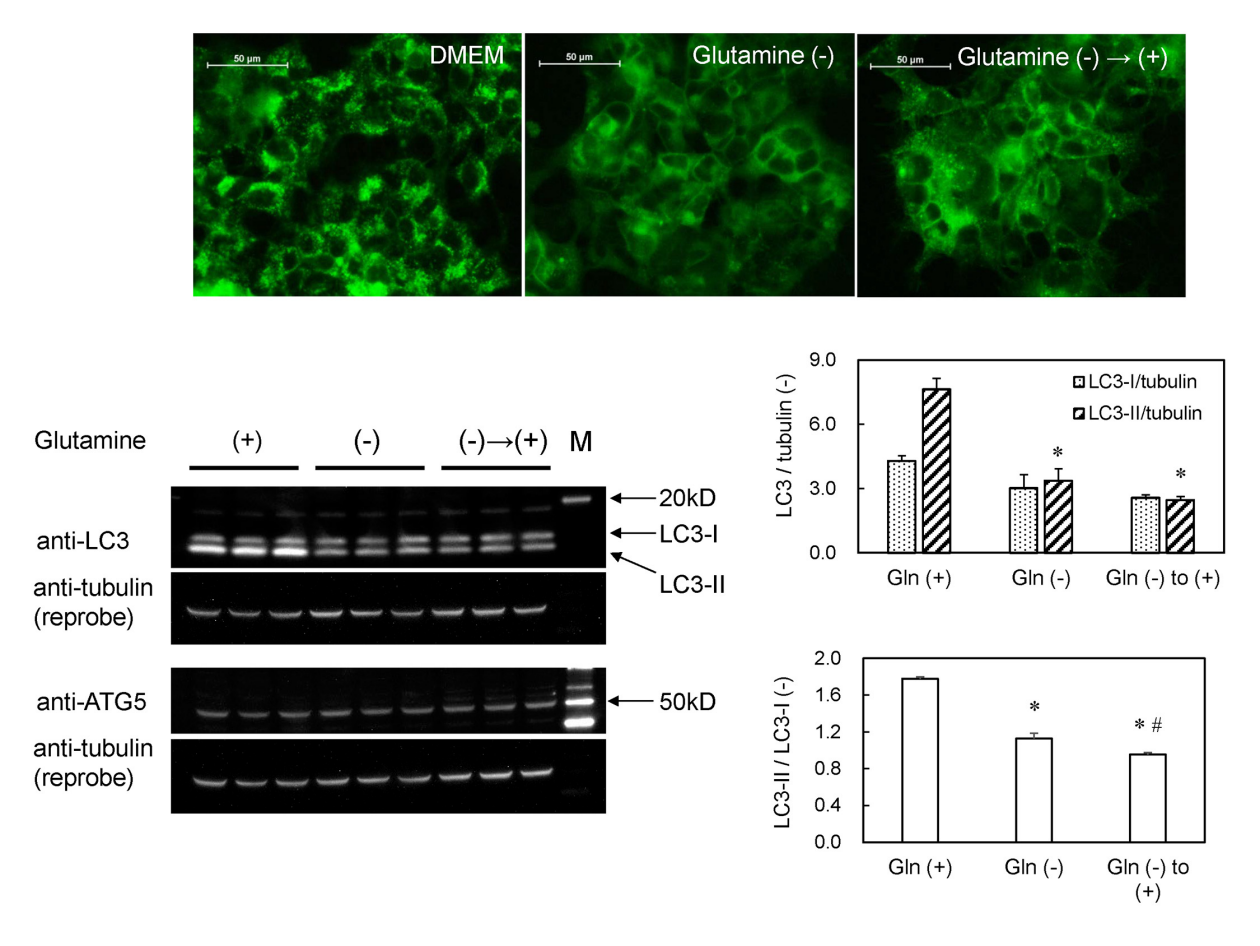
Autophagy in GFP-MARCO-HEK cells is induced by L-glutamine and amines. (A) The cells were cultured (a) in DMEM complete medium for 6 hr; (b) in L-glutamine-free DMEM for 6 hr; or (c) in L-glutamine-free DMEM for 6 hr and then in DMEM (4 mM L-glutamine) for 4 hr. Fluorescent puncta appeared at 4 hr in the presence of 4 mM L-glutamine. The images were captured by fluorescence microscopy. (B) Western blot analysis of LC3 and ATG5 in GFP-MARCO-HEK cells. The cells were cultured in complete DMEM (4 mM L-glutamine) (left three lanes), L-glutamine-free DMEM for 6 hr (middle three lanes), or L-glutamine-free DMEM for 6 hr followed by culturing in complete DMEM (4 mM L-glutamine) (right three lanes). M, Western marker lane. α-Tubulin was adopted as loading control and the amount of LC3-I, LC3-II and tubulin were measured by densitometry. LC3-I/tubulin, LC3-II/tubulin, and LC3-II/LC3-I ratios were presented as mean ± SEM (N = 3). *, Significantly different from glutamine (+). #, Significantly different from glutamine (-).

The autophagic fluorescent small puncta were also observed in GFP-MARCO-HEK cells when these cells were cultured in complete DMEM medium (containing 4 mM glutamine). Those puncta disappeared 6 hr after the medium was replaced with glutamine-free DMEM medium and emerged again 4 hr after the glutamine-free medium was replaced with complete DMEM medium (Fig 7A). The LC3-II was decreased when the cells were cultured in glutamine-free DMEM. However, the LC3-II/LC3-I ratio remained unchanged or even decreased a little after a further culture medium change to complete DMEM for 4 hr (Fig 7B), although the autophagic fluorescent small puncta re-appeared under these conditions (Fig 7A). The level of ATG5 protein, a key molecule of autophagy, was unaltered by any of the medium changes. Those results suggest that the lipidation of LC3 was not requisite for the formation of MARCO-containing autophagic small puncta in GFP-MARCO-HEK cells. Exposure to amines such as methylamine hydrochloride and 2-aminoethanol generated the small fluorescent puncta in GFP-MARCO-HEK cells (S3 Fig).

## Discussion

MARCO is a key molecule that recognizes unopsonized particles in macrophages, although MARCO’s signal transduction cascade upon attachment to particles is unknown. Uptake of polystyrene particles was highly enhanced by transient overexpression of MARCO irrespective of the particle size (20 nm, 200 nm, and 1 μm) [23], suggesting that MARCO functions as a “fly ribbon” on the plasma membrane of macrophages. MARCO is probably processed and digested in the cells, because MARCO was not detected as a single band and many fragments were observed in western blot analysis of GFP-MARCO proteins using anti-MARCO antibody [21]. As we demonstrate in the present work, MARCO was internalized by macropinocytic ruffling of plasma membrane in GFP-MARCO-CHO cells, and the resulting vesicles moved centripetally and disappeared in several minutes. Additionally, we observed that GFP-MARCO-containing macropinocytic vesicles or macropinosomes did not co-localize with lysosomes. The fate of macropinosomes is not well understood. In the current study, there was no sign that macropinosomes fused with lysosomes in GFP-MARCO-CHO cells, suggesting that GFP-MARCO-containing macropinosomes probably were recycled by fusion with the plasma membrane, as previously reported in A431 and 3T3 cells [24]. However, it is possible that part of the macropinocytic membrane in our cells was incorporated into the autophagic small puncta; indeed, phagocytic membrane has been detected in autophagosomes in HeLa cells [25].

Surprisingly, small fluorescent puncta were formed in GFP-MARCO-CHO and GFP-MARCO-HEK cells when the cells were cultured in DMEM. These small puncta were observed only rarely when the cells were cultured in F12 culture or glutamine-free DMEM medium. We compared the composition of F12 with that of DMEM, and noted that a number of components (including pyruvate, vitamins, trace metals such as zinc and copper, putrescine, linoleic acid, and lipoic acid) are present in F12 culture medium and are missing or present only at lower concentrations in DMEM. None of these components abrogated the generation of the small puncta in GFP-MARCO-CHO cells when used to supplement DMEM. In contrast, DMEM contains L-glutamine at a level 4 times higher than that of F12 culture medium (4 mM vs. 1 mM), and culture in F12 supplemented with L-glutamine induced the formation of small puncta in GFP-MARCO-CHO cells. GlutaMAX^TM^ (L-alanyl-L-glutamine), which is gradually hydrolyzed to release L-glutamine, was less effective than L-glutamine; 4 mM ammonium carbonate, a direct ammonia source, induced the puncta rapidly. These results suggest that intracellular ammonia generated by glutaminolysis induced the small puncta when GFP-MARCO-CHO cells were cultured in DMEM. The puncta were not formed in glutamine-free DMEM. However, supplementation of glutamine-free DMEM with 1 mM glutamine (i.e., to the level contained in F12 culture medium) was sufficient to induce the formation of small puncta after 24 hr (data not shown). These results suggest that although glutamine is necessary for the generation of puncta, other medium components modulate the generation and/or stability of the puncta. Intriguingly GFP-MARCO-CHO cells grew faster in F12 than in DMEM, and glutamine supplementation did not further enhance the cell proliferation, suggesting that the small fluorescent puncta were formed in the presence of glutamine regardless of growth conditions.

Macropinocytosis is an actin-dependent process that permits uptake of extracellular fluid and proteins. Endocytic vesicles are continuously formed by invagination and scission of plasma membranes [26]. In the process of endocytosis, plasma membrane pinches off and fuses with ATG16L1 to form phagophores [14]. Two different classes of endosomes (ATG9-associated and ATG16L1-associated) appear to be incorporated into LC3-negative phagophores, with the Soluble NSF Attachment protein REceptor (SNARE) mediating the coalescence of those two different endosomes [27–29]. Our work revealed that the autophagic small fluorescent puncta were still observed even when macropinocytosis was inhibited by cytochalasin D and dynasore inhibited the production of the small puncta, suggesting that the endocytic pathway was the dominant contributor to the generation of MARCO-containing autophagic membranes.

It has been reported that autophagosomes are formed randomly in the peripheral regions in mammalian cells and move centripetally and centrifugally at a speed of 0.1 μm/sec in normal rat kidney cells, gradually distributing in the peri-nuclear region [30]. It also has been shown that the centripetal movement of autophagosomes is dependent on dynein, the microtubule motor protein. In our current study, some small puncta traveled to and fro between the peripheral and peri-nuclear sites at a speed of up to 2–3 μm/sec, although other small puncta appeared to move more slowly. The movement of puncta was impaired upon disruption of tubulin by nocodazole, suggesting that the small fluorescent puncta movement was controlled via microtubules. It has been reported that mTOR is activated and lysosomes are maintained at the periphery of the cell through microtubule-dependent mechanisms when nutrients are sufficient [31]. In contrast, nutrient-deprivation increases intracellular pH from 7.1 to 7.7, resulting in accumulation of lysosomes in the peri-nuclear area and deactivation of mTOR [32]. The lysosomal positioning regulates mTORC1 signaling, which in turn influences autophagosome formation and autophagosome-lysosome fusion rates [32]. The present study suggests that generation of ammonia by exposure of cells to excess glutamine and/or amines increases intracellular pH; intracellular alkalinization presumably impairs lysosomal functions, resulting in accumulation of endocytosis-derived MARCO-containing autophagosomes.

The conversion of LC3-I to LC3-II reflects conjugation of phosphatidylserine and phosphatidylethanolamine to LC3-I [33, 34], a process that is inhibited by protein kinase A-mediated phosphorylation [35]. Glutamine has been shown to increase the levels of LC3-II and autophagosomal puncta in parallel to inactivation of mTOR and MAP p38 in rat intestinal epithelial and human colonic epithelial cells [36]. Glutamine synthetase synthesis is transcriptionally regulated by PI3K-PKB-FOXO signaling, and intracellular glutamine up-regulates autophagy by preventing the translocation of mTORC1 to lysosomal membranes [37]. Those reports suggest that intracellular glutamine induces autophagy via mTOR, a traditional autophagy key molecule that is inhibited by rapamycin and nutrient starvation. In contrast, glutamine also induces autophagy via ammonia [22], a by-product of glutamine metabolism. Glutaminolysis- or ammonia-induced pro-autophagic signals have been reported not to be attributable to nutrient depletion or to inhibition of mTOR [22, 38]. Ammonia, which is generated in mouse embryonic fibroblasts when the cells are deprived of glucose, is known to induce ULK1/2-mTOR-independent and ATG5-dependent autophagy [39]. The present study suggests that glutamine at a typical culture medium concentration (4 mM) is sufficient to induce accumulation of autophagic puncta via ammonia production, and that ammonia is an effective signal for the conversion of LC3-1 to LC3-II.

It has been reported that ectopically expressed MARCO was observed on the cell surface in Jurkat and L929 cells, whereas it was retained intracellularly in J774.1, EL-4, WEHI-3B, and P338D_1_ cells. The different behavior of MARCO in those cell lines may be due to the presence or absence of molecular chaperons required for the correct folding and assembly of the trimeric and functional MARCO [40]. Our current study indicates that MARCO molecules on the cell membrane were continually internalized by macropinocytosis and endocytosis in GFP-MARCO-CHO cells. It remains to be answered whether the dynamic behavior of exogenously overexpressed MARCO in GFP-MARCO-CHO cells is also seen in endogenous MARCO molecules in primary macrophages.

## Conclusions

A plasma membrane receptor MARCO was incorporated into both macropinosomes and endosomes, although only the latter vesicles were further processed into autophagosomes. Ammonia generated from glutamine inhibited autophagy. Although MARCO was first reported to be a phagocytic receptor for environmental particles and microorganisms, the present study suggests that MARCO is internalized during macropinocytic and endocytic processes. It is also possible that the uptake of unopsonized particles via MARCO is brought about significantly by macropinocytic activity of phagocytes, if MARCO functions mainly as a “fly ribbon”.

## Supporting Information

S1 Fig Intracellular processing of GFP-MARCO was hampered by bafilomycin. GFP-MARCO-CHO cells were cultured for 15 hr in fresh F12 medium supplemented with 0.5 μM bafilomycin.A1 Small GFP-MARCO puncta were observed as the late autophagy was inhibited.
(TIFF)

S2 Fig Rapamycin does not affect ammonia-induced generation of autophagic puncta. GFP-MARCO-CHO cells were cultured in F12 complete medium to 50% confluence, and rapamycin was added to concentrations of 0, 100 nM, or 1 μM. After 24 hr, (NH_3_)_2_CO_3_ was added to each well to a final concentration of 0 or 4 mM and the cells were further cultured for 5 hr. The cells were imaged by fluorescence microscopy.
(TIFF)

S3 Fig Effects of amines on autophagic GFP-MARCO puncta generation in GFP-MARCO-HEK cells. The cells were cultured overnight in glutamine-free culture medium and then further cultured for 8 hr in the presence of 8 mM methylamine hydrochloride or 8 mM 2-aminoethanol.
(TIFF)

S1 Movie Confocal microscopic observation (*x-y-t* mode) of CHO-GFP-MARCO cells in glass-bottom culture dishes. Frames were recorded every 5 min while the cells were cultured in an incubation chamber installed over the microscope. The movie was built up at a rate of 3 frames per second.
(WMV)

S2 Movie The CHO-GFP-MARCO cells were cultured in a cell culture plastic dish and incubated with 4 mM (NH_4_)_2_CO_3_ for 15 hr. The movie was taken in real time (5 frames/sec) using an inverted fluorescence microscope. Some vesicles moved quickly from the peri-nuclear region to the distal area of the cells or vice versa. The puncta shown by the arrows moved along the radial direction with a velocity of 2.6 (upper arrow) and 1.9 μm/sec (lower arrow). The scale bar shows 50 μm.
(WMV)
