# Supplementary figures and images for: Macrophage Receptor with Collagenous Structure (MARCO) Is Processed by either Macropinocytosis or Endocytosis-Autophagy Pathway
Source: PLoS One. 2015 Nov 6;10(11):e0142062. doi: 10.1371/journal.pone.0142062 (PMC4636388; doi:10.1371/journal.pone.0142062)

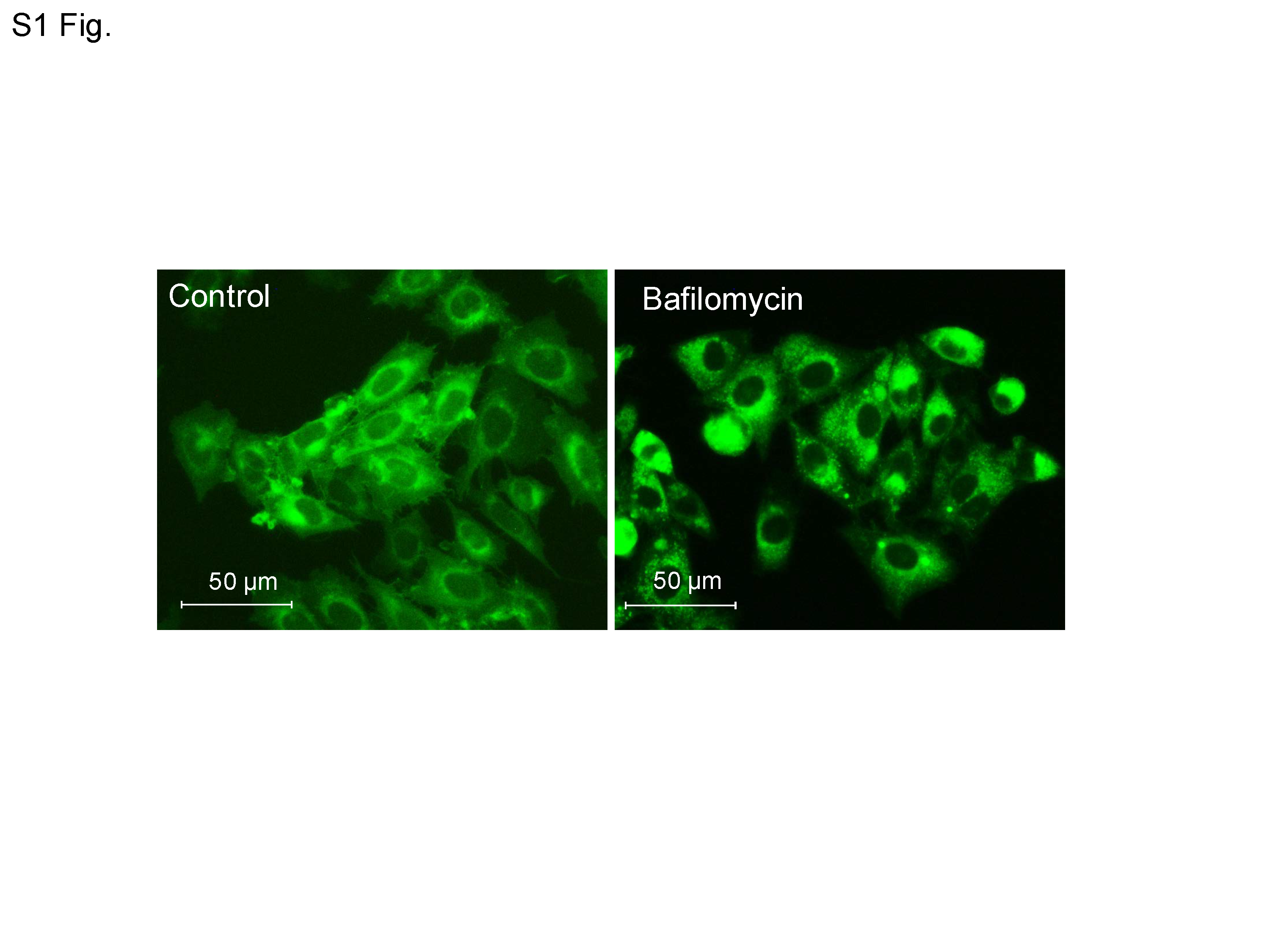

Supplement: S1 Fig — GFP-MARCO-CHO cells were cultured for 15 hr in fresh F12 medium supplemented with 0.5 μM bafilomycin.A1 Small GFP-MARCO puncta were observed as the late autophagy was inhibited. (TIFF) [file pone.0142062.s001.tiff]

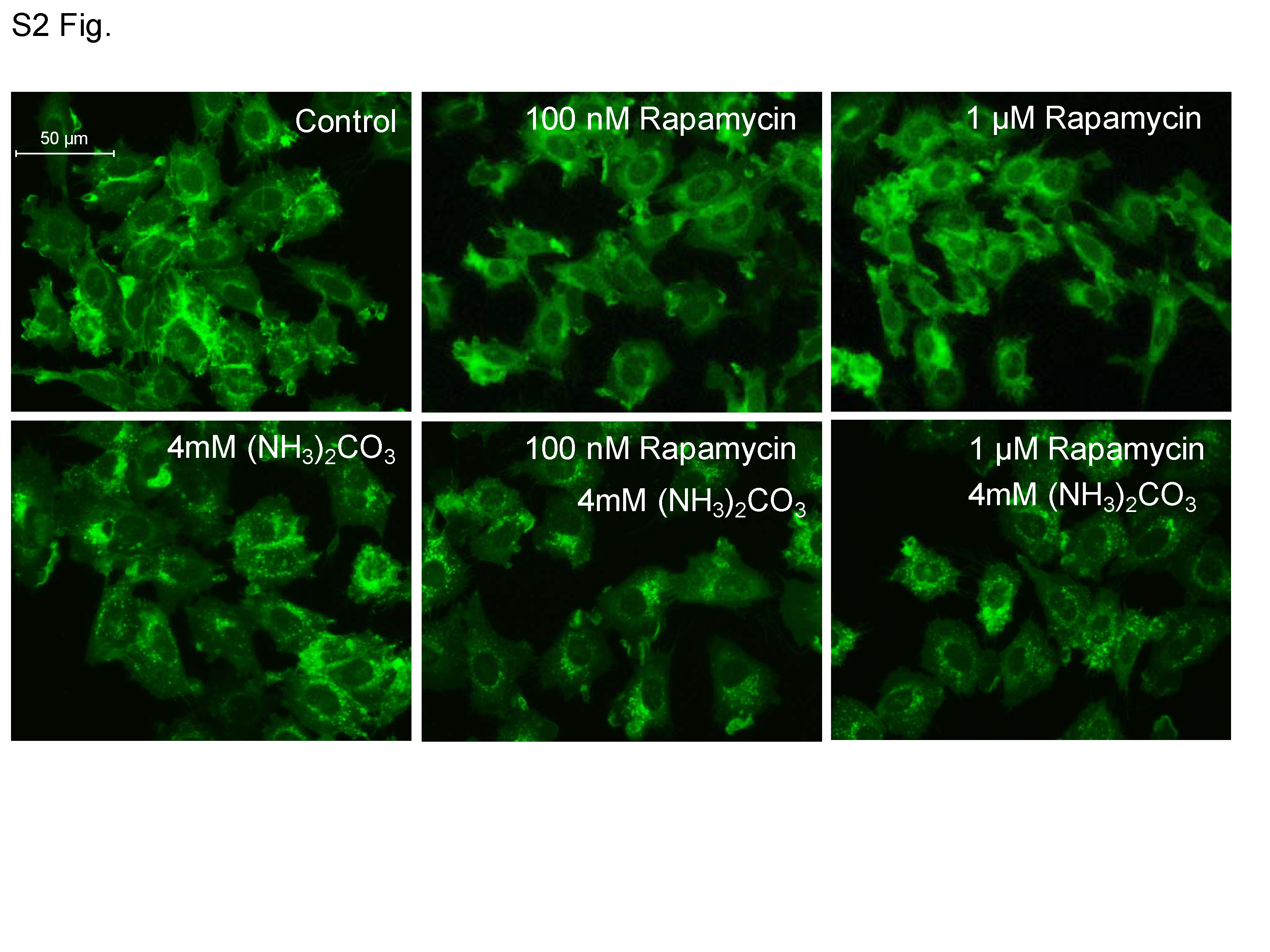

Supplement: S2 Fig — GFP-MARCO-CHO cells were cultured in F12 complete medium to 50% confluence, and rapamycin was added to concentrations of 0, 100 nM, or 1 μM. After 24 hr, (NH3)2CO3 was added to each well to a final concentration of 0 or 4 mM and the cells were further cultured for 5 hr. The cells were imaged by fluorescence microscopy. (TIFF) [file pone.0142062.s002.tiff]

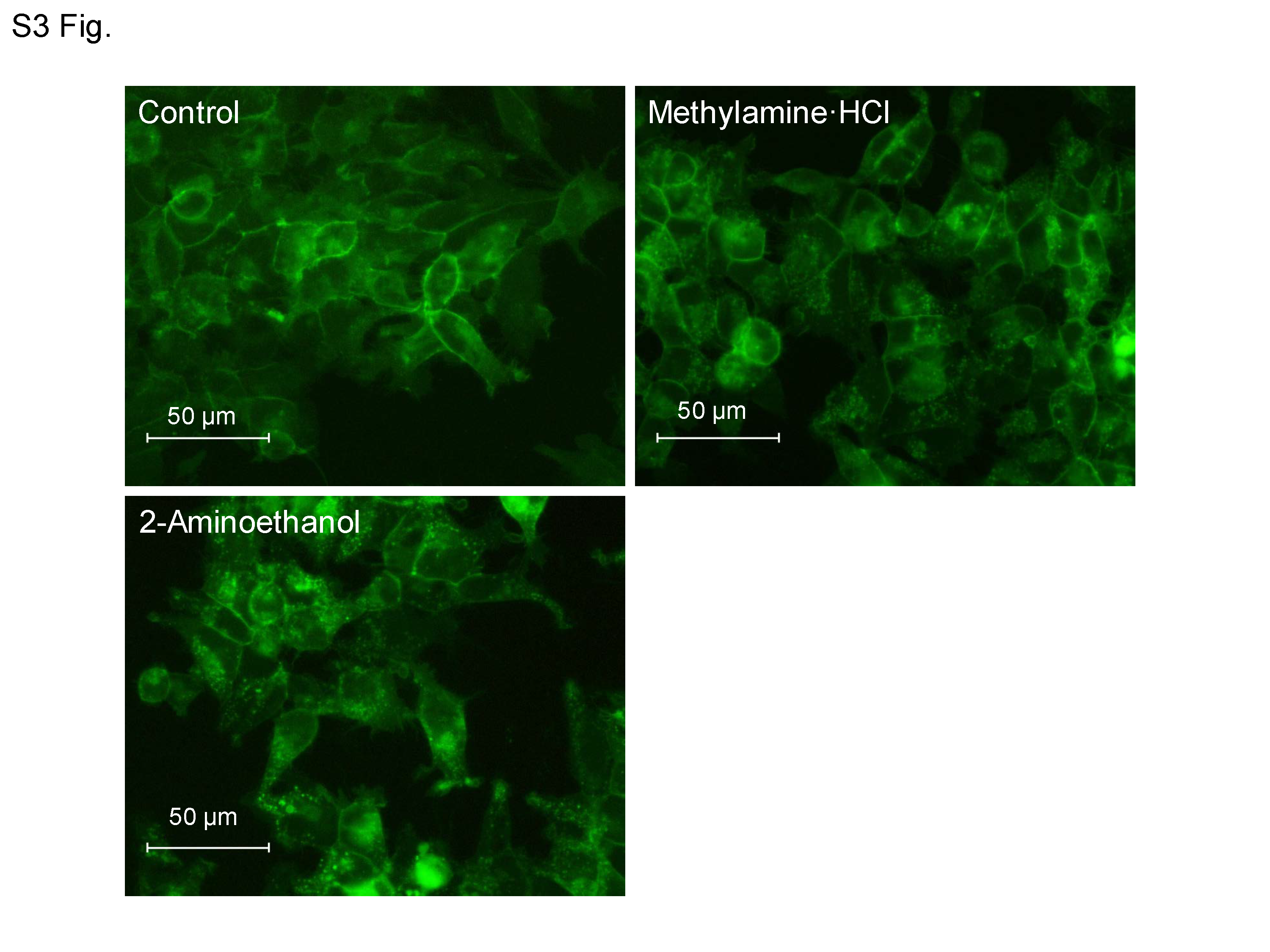

Supplement: S3 Fig — The cells were cultured overnight in glutamine-free culture medium and then further cultured for 8 hr in the presence of 8 mM methylamine hydrochloride or 8 mM 2-aminoethanol. (TIFF) [file pone.0142062.s003.tiff]
